# Epidemiology of Venous Thromboembolism in Belgium: A Cohort Study

**DOI:** 10.1055/a-2688-4768

**Published:** 2025-09-08

**Authors:** Andreas Verstraete, Nicholas Cauwenberghs, Shayan Calhori, Joren Van Durme, Kathleen Freson, Peter Verhamme, Tatiana Kuznetsova, Thomas Vanassche

**Affiliations:** 1 Department of Cardiovascular Sciences, KU Leuven, Leuven, Belgium; 2 Department of Cardiovascular Diseases, University Hospitals Leuven, Leuven, Belgium

**Keywords:** venous thromboembolism, epidemiology, pulmonary embolism, deep venous thrombosis

## Abstract

**Background:**

Venous thromboembolism (VTE) is a significant contributor to global morbidity and mortality. Although management strategies and the distribution of risk factors have evolved, contemporary epidemiologic data are limited and have not been previously reported for Belgium. We aimed to characterize the epidemiology of VTE in a contemporary Belgian population.

**Methods:**

We conducted secondary analyses of 1,448 participants from the Flemish Study on Environment, Genes, and Health Outcomes (FLEMENGHO), an observational, community-based, prospective cohort study. VTE cases occurring between 2000 and 2024 were identified through standardized health questionnaires, medical records, and expert adjudication. The incidence and lifetime risk of VTE were determined, and risk factors for incident VTE were assessed using Cox regression.

**Results:**

Between 2000 and 2024, 63 VTE events occurred during 34,906 person-years of follow-up, corresponding to an incidence rate of 1.80 per 1,000 person-years. At the age of 45, the estimated remaining lifetime risk of VTE was 8.2% (95% CI: 5.6–10.8). Isolated lower extremity deep vein thrombosis was the most common presentation (42.9%), followed by isolated pulmonary embolism (36.5%). Transient major risk factors were identified in 30.2% of cases. In multivariable analyses, higher BMI (adjusted hazard ratio [HR _adj_ ]: 1.48, 95% CI: 1.13–1.93) and a history of VTE (HR _adj_ : 10.4, 95% CI: 4.1–26.3) were independent predictors of incident VTE.

**Conclusion:**

Despite advancements in management strategies, the burden of VTE remained substantial in this representative and well-characterized Belgian cohort. The incidence rate is consistent with findings in other Western countries.

## Introduction

Venous thromboembolism (VTE), encompassing deep vein thrombosis (DVT) and pulmonary embolism (PE), is a common condition that significantly contributes to both short- and long-term morbidity and mortality. VTE is the third leading cause of cardiovascular death, following stroke and myocardial infarction. 1 However, public awareness of VTE remains low when compared with that of myocardial infarction and stroke. 2 Historically, the incidence of VTE in Western countries has been reported to range from 1 to 2 cases per 1,000 person-years. 3 4 5 However, these estimates are based on older cohorts, while the distribution of risk factors and management strategies (e.g., introduction of direct oral anticoagulants) have evolved over time. Moreover, the epidemiology of VTE in Belgium has never been investigated, despite being important for promoting public awareness and for assessing the clinical and economic burden of the disease. Therefore, we aimed to assess the incidence, lifetime risk, and risk factors of VTE in a contemporary Belgian population.

## Methods

### Study Cohort

The Flemish Study on Environment, Genes, and Health Outcomes (FLEMENGHO) is an observational, community-based cohort study on cardiovascular risk factors and outcomes in the adult Belgian population. Between 1985 and 2010, we randomly recruited a family-based population sample, stratified by age and sex, from a geographically defined area in northeastern Belgium, as described previously. 6 From May 2005 to March 2015, 1,851 FLEMENGHO participants were invited to a detailed in-person study visit that comprised standardized and in-depth registration of medical history, lifestyle, medication use, and biochemical profiling. Written informed consent was obtained from 1,448 individuals (participation rate of 78.2%). This subset of 1,448 participants comprised the study sample for the present analysis as the standardized and in-depth data retrieved during the 2005–2015 study visits enabled us to reliably investigate the determinants of incident VTE. The study was approved by the Ethics Committee of the University of Leuven (nr. S64406).

### Assessment of Outcome

Information on diseases, including VTE, was continuously collected from multiple sources until December 2024. At each study visit or phone contact, participants completed a standardized health questionnaire on their medical history. These self-reported diseases were verified and supplemented with information from their general practitioner and from medical health records provided by regional hospitals. Additionally, vital status was obtained from the Belgian Health Registry annually and the immediate and underlying causes of death were obtained. All fatal and non-fatal events were coded using the International Classification of Diseases (ICD) and timestamped. For this study, all VTE-related ICD entries were manually reviewed once more by a senior physician (A.V.) to ensure only clinically evidenced VTE events were included in the analyses. As such, we collected clinically confirmed VTE that occurred in the FLEMENGHO subset until December 2024.

Based on the ICD-coded events, the same senior physician (A.V.) reviewed each VTE case occurring between 2000 and 2024, classifying the type of VTE (DVT, PE, unusual site VTE) and, in case of a DVT, its location (lower or upper extremity). Superficial thrombophlebitis cases were excluded from the analysis. Additionally, he evaluated the etiology of each event, distinguishing between (1) unprovoked VTE (with or without minor risk factors) and (2) provoked VTE associated with a major risk factor, including major surgery, major trauma, active cancer, or pregnancy (either prenatal or <3 months post-partum).

### Other Data

Detailed clinical information was retrieved during the in-person study visit (2005–2015), including body metrics (height, weight, BMI, obesity if BMI ≥30 kg/m ^2^ ), hemodynamics (sitting systolic and diastolic blood pressure and heart rate), and lifestyle factors (alcohol consumption and smoking behavior). Additionally, medication use at the time of the study visit was obtained via a standardized questionnaire. For this study, we focused on the use of antihypertensive drugs, lipid-lowering drugs, anticoagulants, antiplatelet agents, oral contraceptives, and hormonal therapy. On fasting blood samples, a certified laboratory assessed blood sugar levels, creatinine, estimated glomerular filtration rate (eGFR), total cholesterol, LDL cholesterol, HDL cholesterol, and triglycerides.

### Statistical Analysis

Data management and statistical analyses were performed using Python version 3.12. We first estimated overall and age-specific incidence rates of VTE in the study cohort between January 2000 and December 2024. Lifetime risk was then calculated at three index ages—45, 55, and 65 years—in participants without a prior history of VTE at the index age, as the cumulative incidence of VTE up to the age of 80, following previously described methods. 2 7 Remaining lifetime risk estimates were conditional on survival (alive and free of VTE) and accounted for the competing risk of death. 7 Each subject was thus classified according to the first event that occurred: incident VTE, non-VTE-related death, or censoring (due to loss to follow-up or completion of follow-up without event), whichever occurred first. Additionally, we described the type and etiology for the VTE events diagnosed between 2000 and 2024.

Subsequently, we determined risk factors for VTE cases occurring after the in-person study visit (2005–2015). The date of the in-person visit then served as the index date to label incident VTE. We compared the means and proportions in the characteristics of participants with and without incident VTE through unpaired T- and Chi-square testing, respectively. Covariables with *P* < 0.05 were included in a multivariable Cox regression model for prediction of incident VTE. A sensitivity analysis was conducted in participants without prior VTE at the time of the in-person study visit.

## Results

### VTE Incidence and Lifetime Risk

**Fig. 1:**
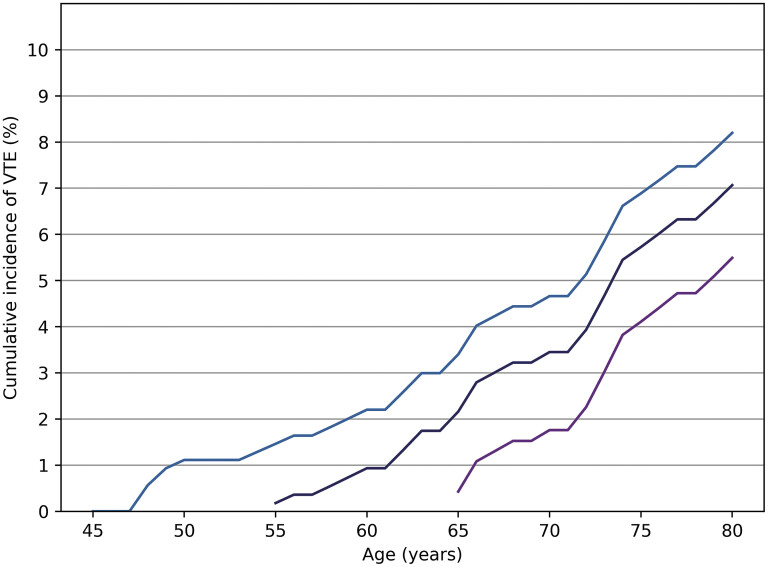
Cumulative lifetime risk of venous thromboembolism (VTE) at selected index ranges in the Flemish Study on Environment, Genes, and Health Outcomes study (FLEMENGHO) sample from 2000 to 2024. Lifetime risk for a given index age—45, 55, or 65 years old—represents the cumulative risk of VTE up to the age of 80 years in participants without a prior history of VTE at the index age. The adjusted cumulative incidence of VTE, accounting for the competing risk of death, was 8.2% (95% CI: 5.6–10.8) for an index age of 45, 7.1% (95% CI: 4.6–9.6) for index age of 55, and 5.5% (95% CI: 3.2–7.8) for index age of 65.

We included 1,448 participants with a mean age of 51.5 ± 15.9 years and a balanced gender distribution (51% women). Participant characteristics at the time of the in-person study visit (2005–2015) are provided in Supplementary Table S1 . Between 2000 and 2024, 63 VTE events were documented during 34,906 person-years of follow-up. The overall incidence rate for the entire follow-up period was 1.80 per 1,000 person-years ( Table 1 ). The incidence rate of VTE increased from 0.83 per 1,000 person-years of follow-up during the 2000–2010 period to 2.49 per 1,000 person-years from 2010 to 2024. Across age groups, the incidence rates of VTE for the entire follow-up period (2000–2024) were 0.76 per 1,000 person-years for participants under 45 years, 1.50 for those aged 45 to 60 years, 3.58 for those aged 60 to 75 years, and 3.02 for those aged 75 years or older ( Table 1 ). When excluding those who experienced a VTE event before 2000 ( *n* = 10), the overall incidence rate was 1.53 per 1,000 person-years. Among participants free of VTE at the age of 45, the estimated remaining lifetime risk of VTE was 8.2% (95% confidence interval: 5.6–10.8). The lifetime risk decreased with increasing age at baseline, reflecting fewer remaining years of life, and consequently, a shorter period at risk among older individuals ( Fig. 1 ).

**Table 1:** Overall and age-specific VTE incidence rate in the FLEMENGHO sample between 2000 and 2024

Age group	Entire cohort ( *n* = 1,448)			VTE-naive cohort ( *n* = 1,438) a		
	# VTE	Person-years	Incidence per 1,000 py	# VTE	Person-years	Incidence per 1,000 py
All	63	34,906	1.80	53	34,716	1.53
<45 years	9	11,800	0.76	9	11,800	0.76
45–60 years	20	13,305	1.50	13	13,230	0.98
60–75 years	28	7,815	3.58	27	7,742	3.49
>75 years	6	1,986	3.02	4	1,944	2.06

Abbreviations: FLEMENGHO, Flemish Study on Environment, Genes, and Health Outcomes study; VTE, venous thromboembolism.

^a^ Participants without a personal history of VTE on January 1, 2000.

### VTE Types and Etiology

The most common type of VTE was isolated DVT of the lower extremity (42.9%), followed by isolated PE (36.5%), concurrent DVT and PE (15.9%), and DVT of the upper extremity (4.8%) ( Supplementary Table S2 ). The majority of VTE events (69.8%) were unprovoked, with 21 of the 44 unprovoked cases occurring in the presence of minor risk factors such as (cast)immobilization, prior infection, or a personal or family history of VTE ( Supplementary Table S2 ).

### Risk Factors for Incident VTE

After the in-person study visit (2005–2015), the cohort was followed-up for a median of 12.9 years (10–90th percentile: 9.2 to 18.1 years; 19,175 person-years), during which 41 VTE events occurred (incidence rate: 2.14 events per 1,000 person-years). Unadjusted analyses showed that participants who experienced a VTE event after the in-person study visit were older (59.0 ± 13.7 vs. 51.3 ± 1 5.9), more likely to be obese (39.0% vs. 19.3%) or hypertensive (61.0% vs. 43.4%), and had a higher prevalence of prior VTE (14.6% vs. 1.1%), use of antiplatelet drugs (24.4% vs. 11.8%), and a higher incidence of cancer (24.4% vs. 12.4%) as compared with those who did not experience a VTE event ( *P* < 0.05 for all) ( Supplementary Table S1 ). In multivariable-adjusted Cox analysis, the risk for incident VTE increased with higher BMI (standardized and adjusted hazard ratio [HRadj] [95% confidence interval]: 1.48 [1.13 to 1.93], *p* = 0.0041) and a personal history of VTE at baseline (HRadj: 10.4 [4.1 to 26.3], *P* < 0.0001) ( Table 2 ). A sensitivity analysis of subjects without a history of VTE at the time of the in-person study visit (2005–2015) confirmed the association between the increased hazard ratio for incident VTE and higher BMI (HRadj: 1.44 [1.07 to 1.94], *p* = 0.015) ( Table 2 ).

**Table 2:** Association between incident VTE and baseline characteristics in a multivariable Cox regression model

Baseline characteristic	Entire cohort ( *n* = 1,448)		No history of VTE at baseline a ( *n* = 1,422)	
	Hazard ratio (95% CI)	*P* value	Hazard ratio (95% CI)	*P* value
Age, per 1 SD increase	1.34 (0.97 to 1.84)	0.074	1.40 (0.99 to 1.96)	0.051
BMI, per 1 SD increase	1.48 (1.13 to 1.93)	0.0041	1.44 (1.07 to 1.94)	0.015
Systolic BP, per 1 SD increase	1.07 (0.80 to 1.44)	0.63	1.18 (0.88 to 1.59)	0.26
History of VTE	10.4 (4.1 to 26.3)	<0.0001	n/a	–
Antiplatelet therapy	1.80 (0.89 to 3.63)	0.10	1.59 (0.73 to 3.49)	0.25
eGFR (CKD-EPI), per 1 SD increase	1.06 (0.82 to 1.37)	0.65	1.05 (0.80 to 1.39)	0.72
Incident cancer	1.57 (0.79 to 3.09)	0.20	1.64 (0.80 to 3.35)	0.17

Abbreviations: BMI, body mass index; BP, blood pressure; eGFR, estimated glomerular filtration rate; n/a, not applicable; VTE, venous thromboembolism.

Note: Covariables were selected based on their significant difference between subjects with and without incident VTE in unadjusted comparison (see Supplementary Tables ). For continuous variables, hazard ratios are presented for a 1 standard deviation (SD) increase in the predictor.

a Sensitivity analysis restricted to participants without a personal history of VTE at the time of the in-person study visit between 2005 and 2015.

## Discussion

In this observational cohort study, we investigated for the first time the epidemiology of VTE in a representative Belgian population sample. Between 2000 and 2024, the overall incidence rate of VTE was 1.80 per 1,000 person-years. The majority of VTE cases occurred in the absence of major risk factors. Higher BMI and a personal history of VTE emerged as the two most significant risk factors.

The incidence rate of VTE observed in our cohort is consistent with rates reported in earlier cohorts and aligns with contemporary estimates from the United Kingdom (1.83 per 1,000 person-years from 2017–2019), Norway (1.53 per 1,000 person-years in 2017), Sweden (1.90 per 1,000 person-years from 2011 to 2018), France (1.84 per 1,000 person-years from 2010 to 2011), and Canada (1.46 per 1,000 person-years from 2002 to 2012). 1 8 9 10 11 Furthermore, we found a remaining lifetime risk of 8.2% at age 45, which is consistent with Bell et al's study, which reported a lifetime risk of VTE of 8.1% among 14,185 middle-aged adults. 2 These findings underscore the significant lifetime risk of VTE in the general population, with approximately 1 in 12 individuals at the age of 45 expected to suffer from VTE during their remaining lifetime. Despite the widespread adoption of novel treatment strategies, including increased use of direct oral anticoagulants, the burden of VTE remains substantial in contemporary cohorts. This highlights the need for vigilance among both patients and clinicians. Further research is needed to optimize primary and secondary prevention strategies to mitigate the burden of VTE.

The incidence rate of VTE in our cohort increased over time (2000–2010 vs. 2010–2024), likely driven by aging of the study cohort, a well-established risk factor for VTE. Although relatively uncommon in young adults (1 in 10,000 persons per year), VTE incidence rises exponentially with age, reaching nearly 1% per year among the very elderly, aged 85 and older. 4 Additional contributing factors for the observed increase may have included changes in diagnostic practices over time, such as the increased use of CT pulmonary angiography. 12 Unfortunately, data on temporal trends in CT pulmonary angiography use in Belgium were not available.

In 44 of 63 (69.8%) VTE events, no major risk factors, such as prior major surgery or active cancer, were identified, suggesting an underlying inherited predisposition to VTE in most patients. Variability in the definition of unprovoked VTE across studies contributes to differences in classification. In the Canadian cohort, which used a similar definition, 55% of VTE events were unprovoked, whereas the broader criteria for provoked VTE in the French cohort, including hormonal therapy and obesity, resulted in 49% being classified as unprovoked. 1 11

Lastly, a prior history of VTE emerged as the strongest predictor of incident VTE, which supports careful decision-making regarding long-term management strategies after a first VTE event.

The findings of the current study should be interpreted in the context of its limitations. First, the relatively low number of VTE events may have limited the statistical power of our multivariable analyses. This may have led to type II error, potentially hiding previously reported risk factors for VTE, and may have influenced the accuracy of the estimated incidence rates. Nevertheless, the incidence rates observed in our Belgian cohort are consistent with those reported in other, larger contemporary Western cohorts. Notably, we have strengthened the robustness and reliability of the outcome data through long-term, detailed, and repeated characterization of our study participants and the use of multiple clinical data sources to validate each VTE event. This contrasts with many large epidemiologic studies that rely on public health and government databases and ICD coding, which are more susceptible to misclassification. Second, although the random recruitment of FLEMENGHO participants in northeastern Belgium minimized selection bias, the study population consisted almost exclusively of Caucasians, which limits the generalizability of our findings to other racial and ethnic groups.

## Conclusion

In conclusion, despite evolving VTE management strategies, the burden of VTE remained substantial in this representative and well-characterized Belgian cohort. The incidence rate is consistent with findings in other Western countries. Larger databases are needed to further elucidate the epidemiology of VTE and to validate risk factors in the Belgian population.

## Notes

**What Is Known About This Topic?**

VTE is a major cause of morbidity and the third leading cause of cardiovascular death. In Western countries, its incidence is estimated at 1–2 per 1,000 person-years, but these estimates are based on older cohorts predating contemporary risk factor profiles and treatment strategies. Lastly, the epidemiology of VTE in Belgium has never been investigated.

**What Does This Paper Add?**

- This is the first study to describe the epidemiology of VTE in Belgium. Between 2000 and 2024, the incidence of VTE was 1.80 per 1,000 person-years, comparable to other contemporary Western countries. The majority of cases (69.8%) occurred in the absence of major risk factors, while higher body mass index and a prior history of VTE emerged as the strongest predictors of recurrent VTE events.
