# Supplementary material for: Epidemiology of Venous Thromboembolism in Belgium: A Cohort Study
Source: TH Open. 2025 Sep 8;9:a26884768. doi: 10.1055/a-2688-4768 (PMC12445332; doi:10.1055/a-2688-4768)
Supplement: Supplementary file 1 — Supplementary Material [file 10-1055-a-2688-4768_26970656.pdf]

Supplementary Table 1. Characteristics at the time of the in-person study visit (2005 -2015) by incidence of VTE.

Characteristic	No incident VTE (n=1407)	Incident VTE (n=41)
Age, years	51.3 ± 15.9	59.0 ± 13.7*
Female sex, n (%)	723 (51.4%)	16 (39.0%)
BMI, kg/m^2	26.4 ± 4.4	29.3 ± 5.8*
Obesity, n (%)	271 (19.3%)	16 (39.0%)*
Hemodynamics		
Systolic blood pressure, mmHg	130.3 ± 17.4	135.9 ± 18.9*
Diastolic blood pressure, mmHg	80.6 ± 9.7	82.5 ± 11.8
Heart rate, bpm	63.9 ± 9.4	62.5 ± 9.9
Lifestyle		
Regular alcohol consumption, n (%)	557 (39.6%)	13 (31.7%)
Smoking behavior		
Active smokers, n (%)	242 (17.2%)	8 (19.5%)
Previous smokers, n (%)	540 (38.4%)	17 (41.5%)
Pack years, years [a]	11.8 (1.2 to 36.8)	15.0 (0.29 to 35.6)
Risk factors and diseases		
Hypertension, n (%)	611 (43.4%)	25 (61.0%)*
Diabetes Mellitus, n (%)	64 (4.5%)	4 (9.8%)
Chronic kidney disease, n (%)	57 (4.1%)	1 (2.4%)
Venous thrombo-embolism, n (%)	16 (1.1%)	6 (14.6%)*
Cardiovascular disease, n (%)	100 (7.1%)	4 (9.8%)
History of cancer, n (%)	80 (5.7)	4 (9.8)
Incident cancer, n (%)	157 (12.4)	10 (24.4)*
Medication		
Anti-hypertensive drugs, n (%)	352 (25.0%)	15 (36.6%)
Lipid-lowering drugs, n (%)	209 (14.9%)	8 (19.5%)
Anti-platelet drugs, n (%)	166 (11.8%)	10 (24.4%)*
Anticoagulant drugs, n (%)	24 (1.7%)	0 (0.0%)
Oral contraception, n (%)	172 (12.2%)	3 (7.3%)
Other hormonal therapy, n (%)	37 (2.6%)	0 (0.0%)
Biochemistry		
Blood sugar, mmol/L	4.82 ± 0.73	4.92 ± 0.51
Creatinine, mmol/L	79.8 ± 16.7	84.3 ± 15.0

TH Open | DOI 10.1055/a-2688-4768 | © The Author(s).

eGFR (CKD-EPI), ml/min/1.73 m^2	90.8 ± 17.8	84.4 ± 14.3*
Total cholesterol, mmol/L	5.08 ± 0.95	5.14 ± 1.07
HDL cholesterol, mmol/L	1.46 ± 0.38	1.37 ± 0.34
Triglycerides, mmol/L	1.05 (0.61 to 2.12)	1.25 (0.73 to 2.19)

Values are mean +/- standard deviation, median (25-75% percentile) or count (%).
[a] Pack year statistics included current and past smokers.
[*] *P* value < 0.05 for group comparison.
BMI indicates body mass index; CV, cardiovascular; eGFR, estimated glomerular filtration rate; HDL, high-density lipoprotein; n/a, not applicable; VTE, venous thromboembolism.

Supplementary Table 2. Types and etiology of VTE cases in the FLEMENGHO sample between 2000 and 2024.

VTE type	Unprovoked VTE [a]	Provoked VTE [b]	Total
DVT lower extremity	19 (30.2%)	8 (12.7%)	27 (42.9%)
DVT upper extremity	0 (0%)	3 (4.8%)	3 (4.8%)
DVT lower extremity + PE	10 (15.9%)	0 (0%)	10 (15.9%)
PE without DVT	15 (23.8%)	8 (12.7%)	23 (36.5%)
**Total**	44 (69.8%)	19 (30.2%)	63 (100%)

[a] Unprovoked VTE was defined as VTE occurring without major risk factors, including VTE without apparent risk factors (n=23) or with only minor risk factors such as (cast) immobilization (n=4), minor trauma (n=2), recent COVID-19 infection (n=2), recent erysipelas (n=1), recent oral contraception (n=1), and personal (n=10) or family history of VTE (n=1).
[b] Provoked VTE was defined as VTE occurring in the presence of major risk factors including major vascular, oncologic, orthopedic and neurosurgery (n=5), major trauma (n=2), active cancer (n=11), or pregnancy (prenatal or less than 3 months post-partum) (n=1).

DVT indicates deep venous thrombosis; PE, pulmonary embolism; VTE, venous thromboembolism.

TH Open | DOI 10.1055/a-2688-4768 | © The Author(s).
